# Does Entrepreneurship Education Affect Pre-start-up Behavior in Malaysia? A Multi-Group Analysis Approach

**DOI:** 10.3389/fpsyg.2022.738729

**Published:** 2022-02-15

**Authors:** Nor Hafiza Othman, Norasmah Othman, Noor Hasni Juhdi

**Affiliations:** ^1^Faculty of Entrepreneurship and Business, Universiti Malaysia Kelantan, Kelantan, Malaysia; ^2^Faculty of Education, Universiti Kebangsaan Malaysia, Selangor, Malaysia; ^3^Faculty of Economic and Management, Universiti Kebangsaan Malaysia, Selangor, Malaysia

**Keywords:** entrepreneurship education, pre-start-up, business program, non-business program, entrepreneurial behavior

## Abstract

This study investigates the moderating effect of students’ programs on entrepreneurship education aimed at pre-start-up and its effect on the students’ behavior. This study also attempts the level of entrepreneurship education and pre-start-up behavior among students. A survey was carried out among 441 final year students, including 214 students from business programs, and 227 students from non-business programs. Data were analyzed using IBM SPSS 22 and SmartPLS 3.3.0, to perform descriptive and multi-group analysis (MGA), including assessment of measurement invariance of the composite model (MICOM). The results reveal that all direct relationships were supported. It was also found that student programs do have a moderating effect on the relationship between entrepreneurship education and pre-start-up behavior. Furthermore, the results discovered that risk control is a crucial component of entrepreneurship education and should be highlighted in the curriculum. This study contributes to the literature by considering student programs as a moderator, a comparatively new factor in the pre-start-up behavior among university students at the tertiary level. Therefore, entrepreneurship education must be properly designed, and the co-curriculum must be properly organized, so that entrepreneurship will be the preferred career choice in the future.

## Introduction

Entrepreneurship is a gaining much traction in today’s time, when the chance of getting a job in the government and the private sector is on a decline. In this regard, the government emphasizes the development of entrepreneurial skills at all levels of education and training to help create an entrepreneurial community. Moreover, entrepreneurship is considered as an immediate solution to the problem of unemployment, especially among graduates (Jones et al., 2017; Olokundun et al., 2018; Wardana et al., 2020). Entrepreneurship education has thus, been implemented by governments as a strategy through the Third Outline Perspective Plan (OPP 3) with the aim of increasing the number of skilled human resources capable of developing innovation and technology (Othman and Othman, 2019). Therefore, the government introduced the National Entrepreneurship Policy on 9 May 2019 as a guide to provide a holistic framework or ecosystem for entrepreneurship development in Malaysia (Ministry of Entrepreneur Development, 2019).

According to Jones and English (2004), entrepreneurship education is a process that not only teach students how to recognize commercially viable opportunities, but also how to create vision and identity, as well as acquire knowledge and skills so that they can act on them. This means that having a good understanding of the market makes it easier for students to see the entrepreneurial opportunities that exist in their surroundings. While, organizations need workers with leadership qualities who can not only comprehend the market but also provide new value to clients in order to assure organizational growth (Gozun and Rivera, 2017). Intrapreneurs are those who have entrepreneurial qualities including being innovative, proactive, autonomous, risk-taking, self-assured, and so on (Hastuti et al., 2016; Blundel et al., 2018). As a result, it’s not surprising that intrapreneurs in organization are in high demand. Therefore, exposing students to entrepreneurship education can help them acquire not only entrepreneurial skills and knowledge, but also entrepreneurial qualities and attributes.

Entrepreneurship education is implemented and developed through the curriculum of formal and informal teaching and learning activities (Aldianto et al., 2018; Othman and Othman, 2019; Hessels et al., 2020). Thereby, higher education institutes play an important role in ensuring that entrepreneurship education develops the potential of students as well as encourages entrepreneurial activities and subsequently helps them choose an entrepreneurial career over others. Since 2013, all students at higher education institutions in Malaysia, regardless of their field of study, have been required to take an entrepreneurship course. Universities might make entrepreneurship education a compulsory, core, or elective course in the past. In today’s Malaysia, entrepreneurship education has undergone significant changes and has become a national priority.

However, the number of students or graduates starting businesses is still less encouraging, despite various initiatives and incentives given by the government (Ahmed et al., 2017; Koe et al., 2018). According to a report released by UNIRAZAK (2015), the percentage of individuals in Malaysia opting for business start-ups is low at 5.9% compared to other ASEAN countries such as Indonesia (14.2%), Thailand (23.3%), Singapore (11%), Vietnam (15.3%), and the Philippines (18.4%). This needs to be considered more closely, especially those start-ups established via entrepreneurship education because of the current uncertain and increasingly challenging economic situation. Furthermore, the COVID pandemic has also stifled economic growth and sustainability in business. This situation has forced potential entrepreneurs to be bolder in calculating business risks and to explore offbeat business opportunities and subsequently implement entrepreneurial activity in order to start a business.

Moreover, research that has highlighted the behavior of business start-up among students in higher education is very limited and poorly explored, as previous research has focused more on entrepreneurial intention at the tertiary level (Nabi et al., 2017; Othman and Othman, 2019). To achieve economic growth and minimize unemployment in the country, students and graduates must be involved in entrepreneurship. Indirectly, this study provides further empirical evidence on behavior of students who are in the process of starting a business at the university. This is in line with the government’s aspiration to make Malaysia an entrepreneurial nation by 2030 (Ministry of Entrepreneur Development, 2019). Therefore, the exposure to entrepreneurship education is significant in developing potential entrepreneurs starting from being unaware of entrepreneurship, to being able to respond to business start-up activities, and then becoming a job creator.

The rest of the research is organized as follows. The following section covers entrepreneurship education, pre-start-ups behaviors, and the role of student programs at universities, as well as theory and hypotheses related to these topics. The sampling, measurement, and data analysis are covered in the methodology section. The section of the findings that describes descriptive and inferential statistics follows that. It wraps up with a discussion, implications, limitations, and recommendations for future research, as well as conclusions.

## Theory and Hypotheses Development

### Entrepreneurship Education and Pre-start-up Behavior

Entrepreneurship education has a positive impact on the entrepreneurial development of students, as well as increasing their potential to choose entrepreneurship as a career (Jones et al., 2017; Almahry et al., 2018; Moreno et al., 2019; Gieurea et al., 2020). According to Jones and English (2004), entrepreneurship education is one of the processes that can not only provide individuals with the skills to detect opportunities of commercial value, but also cultivate vision and identity. They added that entrepreneurship education aids in developing entrepreneurial knowledge and skills to create opportunities in the business environment. Further, entrepreneurial policy is especially essential for developing entrepreneurial intention after undergoing entrepreneurship education (Huang et al., 2021). Thus, early exposure can enhance students’ entrepreneurial abilities, and in turn influence them to choose an entrepreneurial career.

According to Hytti and O’Gorman (2004), there are three main objectives that will influence the approach and technique of entrepreneurship education programs available to students. If the objective of entrepreneurship education is to improve understanding of the subject, lectures or seminars are a good place to start. Meanwhile, if the objective of entrepreneurship education is to enable students to develop entrepreneurial skills in the workplace, the optimal teaching strategy is to expose students to hands-on training. If the objective of entrepreneurship education is to develop a large number of entrepreneurs, the best teaching strategy is to provide students hands-on experience with entrepreneurship through simulations or role models. As a result, appropriate teaching methods must be highlighted by all parties in order to improve the percentage of graduates selecting entrepreneurship careers.

In this study, researchers want to determine whether students are more capable of dealing with entrepreneurial activities after taking entrepreneurship courses. Hence, entrepreneurship education is referred to as the process of developing students’ entrepreneurial abilities after attending an entrepreneurship learning. According to Peschl et al. (2020) as well as Sanchez and Sahuquillo (2018), entrepreneurial abilities is considered important in encouraging entrepreneurial activities to be carried out. This is in line with the widespread acceptance of the concept of capability as a function of the second type of knowledge, namely “know-what” and “know-how” (Huitt, 2011). So, entrepreneurship education is a significant contributor to the development of students towards entrepreneurship because it can enhance entrepreneurial knowledge and skills as well as encourage them to engage in entrepreneurial activities, thereby choosing entrepreneurial career as a career of choice (Jones et al., 2017; Kuratko and Morris, 2017; Nabi et al., 2017). Thus, higher education institutions play an important role, not only as repositories of knowledge, but also in developing the potential of students to become entrepreneurs.

Besides that, entrepreneurial behavior can be defined as actions leading an individual to the setting up of a new business (Kalufya et al., 2015; Kautonen et al., 2015), encompassed in the term ‘pre-start-up’ or ‘start-up processes’ (Davidsson, 2008; Reynolds, 2016). These actions or activities include surveying suitable locations, raising capital, writing business plans, attending classes, workshops or seminars, gathering information, obtaining funding, applying for licenses or patents, purchasing equipment, and so on (Carter et al., 1996; Jiang and Wang, 2014; Reynolds, 2016; Kuckertz et al., 2017). Some researchers state that a person’s entrepreneurial actions can determine the extent to which they are involved in entrepreneurship (Gartner et al., 2010; Bird et al., 2012). According to Kautonen et al. (2015), the more entrepreneurial activities performed in the start-up process, the more potential the individual has to become an entrepreneur. This study refers to the pre-start-up behavior as a process of action or deed that has been done towards the establishment of a business.

Based on Miltenberger (2001), behavior can be influenced systematically by events or circumstances that occur in the environment that an individual experiences. He explained that the behavior can be demonstrated overtly or covertly, and that these changes depend on their environment. This situation reflects that an entrepreneur does not simply look at and identify opportunities, but also seizes the opportunity to create a business. Moreover, behavioral changes can occur through stimuli and responses arising from the impact of the environment, education, and experience (Vargas, 2015). Indirectly, this shows that entrepreneurship education does affect the behavior of students in the process of starting a business. Appendix 1 provides a table of literature review.

Moreover, the pre-start-up is to identify the extent to which activities are likely to be performed by the potential entrepreneurs during the start-up phase (Elena, 2014; Mamun et al., 2017). Surveying appropriate locations, raising funds, developing business plans, attending classes, workshops, or seminars, gathering information, acquiring funding, filing for licenses or patents, and so on are examples of such activities (Carter et al., 1996; Reynolds, 2016; Kuckertz et al., 2017). Furthermore, when starting a business, the product to be offered is still unknown in the market and thus, various activities or actions need to be considered before operations open. Several researchers explain the importance of preparing a business plan before starting a business (Blank, 2013; Shepherd et al., 2015; Peschl et al., 2020). Blank (2013) explains that by drafting a business plan, individuals are able to predict what might happen in advance, before they implement their ideas.

This study considers the theoretical basis of the human capital theory. Human capital theory explains that students with higher levels of input toward entrepreneurship will produce superior output (Davidsson and Honig, 2003; Werner and DeSimone, 2011). A student with high human capital will be better able to apply entrepreneurial knowledge and skills as well as act on the entrepreneurial activities required in the business start-up process. Shane and Venkatraman (2000) acknowledge that human capital can enhance an individual’s ability to discover and exploit business opportunities that are crucial in entrepreneurial activity and be more sensitive to opportunities that others may not be aware of Ucbasaran et al. (2010), Saenz et al. (2019). Therefore, human capital theory can determine that the higher the students’ entrepreneurial ability after pursuing entrepreneurship education, the higher their potential to engage in entrepreneurial activities. Therefore, the following hypothesis is developed:

H1: Entrepreneurship education has a significant effect on pre-start-up behavior among students

### The Role of Students Program on the Relationship of Entrepreneurship Education and Pre- start-up Behavior

Entrepreneurship education plays an important role in enhancing students’ ability to carry out entrepreneurial activities when starting a business. Moreno et al. (2019) explain that students’ ability to perform business activities greatly influences them to start a business, thus proving that students will be more confident in choosing an entrepreneurial career once they have been exposed to entrepreneurship education. As a result, people who are more confident in their abilities are better able to cope with uncertainty (Koellinger et al., 2007). Indirectly, students or potential entrepreneurs will be more active in the pre-start-up behavior (Mamun et al., 2017; Aldianto et al., 2018; Wardana et al., 2020). Therefore, entrepreneurship education needs to be planned, and the co-curriculum needs to be coordinated and organized carefully to ensure that students choose an entrepreneurial career in future.

Besides, entrepreneurship education or programs can help students to develop entrepreneurial skills and become job creators by creating graduates with entrepreneurial qualities (Lee et al., 2018; Middleton et al., 2019; Ward et al., 2019; Othman et al., 2020). This view is in line with Sequeira et al. (2007) and Bandura (1997) that the perception of self-efficacy, that is, aspects of vocational skills and entrepreneurial knowledge, is fundamental in starting a business. It is not surprising that the practical aspects of entrepreneurship education are play a crucial role in improving students’ entrepreneurial skills. Thus, students or potential entrepreneurs will be more active in entrepreneurial activities in the start-up process. In addition, the entrepreneurs’ success depends significantly on their ability or capability to find, see and take advantage of the opportunities available (Kuckertz et al., 2017; Lundmark et al., 2017). According to Karimi et al. (2016), most current entrepreneurship curricula focus on theoretical expertise rather than practical skills, which prevents students from exploring future opportunities. Thus, a person who wants to succeed must be sensitive to and seize the entrepreneurial opportunities that exist around, before they are grabbed by others.

Despite this, several studies state that formal educational programs do not have a positive impact on students’ entrepreneurial development (Maresch et al., 2016; Mahendra et al., 2017). The perceptions of entrepreneurship can vary between business and non-business students. Business students, according to Galloway and Brown (2002), have a more optimistic outlook and have a firm desire from the start to be a part of the business or industry, as they are more self-assured and capable of becoming entrepreneurs, owing to their knowledge of the ins and outs of business. Therefore, when people continue to believe in their abilities to operate a business, they begin to value themselves favorably in the scope of entrepreneurship.

Students with non-business majors, such as science or architecture, may, contrastingly, have fewer optimistic attitudes and aspirations to become entrepreneurs than students with business majors (Galloway and Brown, 2002). According to the European Commission (2008), non-business students do not concentrate on business subjects because they are preoccupied with other things, and thus have a having a lower understanding of business. Indirectly, it can also distinguish individuals who have entrepreneur and non-entrepreneur characteristics. However, few studies have investigated how student programs affect the relationship between entrepreneurship education and pre-start-up behavior. Thus, the following hypothesis is developed:

H2: Students’ programs will moderate the relationship between entrepreneurship education and pre-start-up behavior.

**FIGURE 1:**
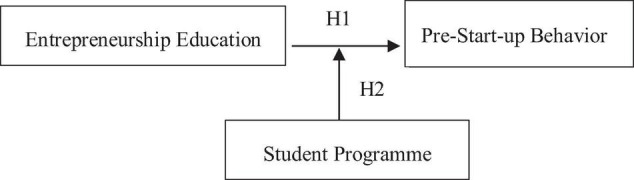
Research model.

Based on the literature review, this study had four research questions, as well as two research hypotheses. Figure 1 shows the developed research model.

## Methodology

### Data and Sample

The data was collected from final-year students using a self-administered questionnaire in Mac 2019 to June 2019. The total population of this study area was 89,349 final year students of first degree public universities (Ministry of Higher Education, 2017). The determination table introduced by Krejcie and Morgan (1970), stipulates that the minimum sample size for a population of over 100,000 people is 384. Thus, a total of 518 questionnaires were distributed to ensure that the data obtained were sufficient in the event of incomplete or lost questionnaires.

Overall, all questionnaires provided to the respondents were collected successfully. Nevertheless, there were seven incomplete questionnaires, and 70 questionnaires had outliers after the data were examined. This shows that there were 441 data sets complete and usable questionnaires, at the return rate of 85.14%, exceeding the target of 80%, as suggested by Cohen et al. (2007). Thus, the survey sample consisted of 441 questionnaires.

Additionally, Harman’s single factor test was used to measure common method variance (CMV) because all the data were gathered from a single source. The results showed that the variance value of the first factor was 42.14%. Thus, there was no CMV problem in this study because the value was less than 50%, as suggested by Podsakoff et al. (2003).

### Measures

The questionnaire was divided into three main parts (see Table 1). Part A consisted of questions pertaining to the respondent’s profile. Part B was regarding entrepreneurship education and was adapted from Lackeus (2015) and Othman and Othman (2017). All the items were measured on ten-point scale ranging from 1 (not at all) to 10 (very high). Meanwhile, ten items were constructed in Part C to measure the pre-start-up behavior using a seven-point scale (1 = strongly disagree, 7 = strongly agree). The statements were adapted from Kuckertz et al. (2017) and Reynolds (2016).

**TABLE 1:** Section of questionnaire.

Parts	Items number
Part A (Demographic)	4
Part B (Entrepreneurship education)	11
Part C (Pre-start-up behavior)	10

To verify the content and face validity, the questionnaire was evaluated by eight experts in entrepreneurship and entrepreneurship education. Once the questionnaire was validated, a pilot study was conducted with 195 final-year students. Next, Cronbach’s alpha values for each construct were observed, that is, entrepreneurship education (0.967) and pre-start-up behavior (0.963). Therefore, these items had good internal stability and consistency when the Cronbach’s alpha value exceeded 0.6 (Nunnally and Bernstein, 1994).

### Data Analysis

This study included both descriptive and inferential statistics. Using the IBM SPSS 22 software, researchers analyzed descriptive statistics by interpreting mean scores and standard deviations from formula Davies (1971). According to him, the target level is divided by subtracting the highest mean score from the lowest mean score. As a result, the interpretation of the mean score used to identify the level of entrepreneurship education is 1.00 to 2.80 displays a very low level, 2.81 to 4.60 suggests a low level, 4.61 to 6.40 indicates a moderate level, 6.41 - 8.20 indicates a high level, and 8.21 - 10.00 indicates a very high level. As for the level of pre-start-up behavior, the mean score value between 1.00 and 2.20 indicates a very low level, 2.21 to 3.40 indicates a low level, 3.41 to 4.60 indicates a moderate level, 4.61 to 5.80 indicates a high level and 5.81 to 7.00 indicates a very high level.

While, to analyze inferential statistics, i.e., moderation through relationships in a research model, this study used multi-group analysis (MGA) with partial least squares path modeling (PLSPM) using SmartPLS 3.3.0 software. When the groups are known, the MGA allows researchers to test for differences between them in two similar models (Hair et al., 2017). Additionally, measurement invariance was performed prior to evaluating the MGA using the composite model measurement invariance of the composite model (MICOM).

A two-stage analytical method recommended by Hair et al. (2017) was used in this study. The structural model assessment was performed after the measurement model assessment. The validity and reliability of the measurement model were evaluated as part of the theoretical model’s assessment using Smart PLS. The structural model was then estimated in terms of in-sample explanatory power (R^2^), out-of-sample predictive relevance (Q^2^), and the significance of the standardized path coefficients, as well as the model fit using standardized and root mean square residuals (SRMR). Then, using Henseler’s MGA and the permutation test, the MGA was performed.

## Results

The results in this section covered the profile of respondents, as well as the use of IBM SPSS 22 to analyze descriptive data and PLSPM analysis to evaluate the hypotheses that had been developed. The majority of respondents were female (62.59%), with 37.41% being male. Comprehensive University had the largest proportion of respondents (37.41 percent), followed by Focused University (34.47 percent), and Research University (28.12 percent). In terms of student programs, 48.53 percent of respondents took the business program, while 51.47 percent took the non-business program. Following that, the most of respondents stated that they would choose to work for a salary rather than self-employment after graduation. This study, however, shows that the percentage of students who opt to work for a wage rather than be self-employed is small, at only 11.2 percent.

### Descriptive Statistics

This section discusses two (2) research questions, specifically level of entrepreneurship education and pre-start-up behavior.

Table 2 displays the mean score of the level of entrepreneurship education among students. Findings show that the level of entrepreneurship education is at a moderate level with a mean value of 5.58. Indirectly, these findings show that entrepreneurship education has a positive impact on students’ self-development towards entrepreneurship. Therefore, with the implementation of entrepreneurship education at the tertiary level, it can increase the ability of students to detect opportunities, obtain information and skills to facilitate them to exploit entrepreneurial opportunities and further venture into the field of entrepreneurship.

**TABLE 2:** Mean and standard deviation of entrepreneurship education.

Construct	Mean	Standard deviation	Interpretation
Entrepreneurship Education	5.58	1.211	Moderate

Following that, a detailed analysis of each items in entrepreneurship education (Table 3). The mean value for each items varied from 4.97 (lowest mean value) to 6.17 (highest mean value). The findings showed that the statements with the highest mean value were “identifying business ideas”, followed by “identifying any changes that occur in the environment” and “relating business ideas with community needs”. While the statement that got the lowest mean was “calculating the business risk”.

**TABLE 3:** Items of entrepreneurship education.

Items		Mean	Standard deviation
A1	able to identify business ideas	6.17	1.431
A2	able to generate innovative business ideas	5.66	1.371
A3	able to identifiy any changes that occur in the environment	5.91	1.432
A4	able to relate business ideas with community needs	5.82	1.366
A5	able to build networking in the business	5.61	1.442
A6	able to prepare a business plan	5.52	1.307
A7	able to prepare financial reports (e.g., cash flow statement, balance sheet)	5.53	1.325
A8	able to calculate the cost of production of goods or services produced	5.47	1.300
A9	able to conduct market research	5.47	1.321
A10	able to evaluate profitable business models	5.24	1.317
A11	able to calculates business risk	4.97	1.236

Next, Table 4 shows the interpretation of mean scores for pre-start-up behavior. The findings indicate that student behavior during the pre-start-up is moderate level. In general, students that are interested in entrepreneurship will engage in entrepreneurship-related activities on their own. As a result, they have made the necessary preparations and efforts to establish a firm.

**TABLE 4:** Mean and standard deviation of pre-start-up behavior.

Construct	Mean	Standard deviation	Interpretation level
Pre-start-up behavior	4.14	1.159	Moderate

The detailed analysis of the pre-start-up behavior items as in Table 5. The mean value for each items varied from 3.91 (lowest mean value) to 4.39 (highest mean value). It was found that the mean value for the highest statements were “I often look for information about new products to start a business”, followed by “I often observe the environment to identify potential business opportunities to venture into” and “I have been exploring new markets that can be explored to start a business”. Thus, this demonstrates that students are making efforts and engaging in activities related to launching a business.

**TABLE 5:** Items of pre-start-up behavior.

Items		Mean	Standard deviation
B1	often observe the environment to identify potential business opportunities to venture	4.29	1.478
B2	often look information about new products to start a business	4.39	1.231
B3	do market research to identify potential business opportunities	4.25	1.265
B4	explore new markets that can be explored to start a business	4.26	1.198
B5	found ways to improve the products or services available in the market	4.22	1.268
B6	prepared a business plan to start a business	4.01	1.337
B7	saved up money to start a business	3.95	1.418
B8	already have a work team to start a business	3.96	1.331
B9	looking at some strategic locations to start a business	3.91	1.407
B10	planned the type of business that will venture	4.19	1.387

### Inferential Statistics

#### Measurement Model Assessment

Construct validity (discriminant validity and convergent validity) and construct reliability of the measurement model were assessed. Factor loadings, average variances extracted (AVE), and composite reliability (CR) were used to determine convergent validity. As described by Hair et al. (2014), all items had factor loadings above 0.70, indicating that all items were acceptable. When the value obtained for each construct exceeded 0.50, the AVE value was achieved (Hair et al., 2017; Awang et al., 2018). The composite reliability (CR) was determined by considering the CR value, which were all greater than 0.60. As shown in Table 6, the measurement models for the full and split datasets were successfully met, resulting in adequate convergent validity.

**TABLE 6:** Assessment of full measurement model and samples.

Construct	Full dataset (*n* = 431)				Business (*n* = 214)			Non-business (*n* = 227)		
	Items	Loadings	CR	AVE	Loadings	CR	AVE	Loadings	CR	AVE
Entrepreneurship education	A1 A2 A3 A4 A5 A6 A7 A8 A9 A10 A11	0.909 0.888 0.891 0.907 0.907 0.892 0.898 0.904 0.882 0.901 0.890	0.969	0.757	0.908 0.904 0.894 0.914 0.913 0.884 0.891 0.903 0.891 0.904 0.894	0.979	0.810	0.897 0.854 0.882 0.875 0.883 0.882 0.891 0.889 0.85 0.882 0.871	0.974	0.771

Pre-start-up behavior	B1 B2 B3 B4 B5 B6 B7 B8 B9 B10	0.877 0.809 0.802 0.876 0.896 0.901 0.89 0.871 0.892 0.878	0.978	0.805	0.899 0.871 0.832 0.878 0.889 0.896 0.912 0.886 0.895 0.886	0.973	0.783	0.844 0.707 0.751 0.857 0.894 0.905 0.847 0.831 0.874 0.856	0.959	0.703

The discriminant validity of this study was determined using the Fornell and Larcker (1981) criterion and the heterotrait-monotrait ratio of correlations (HTMT). When the square root of the AVE of all constructs, as reflected by the values on the diagonals, was higher than the corresponding row and column values, discriminant validity was achieved. The approach claims that discriminant validity between constructs has been defined if the HTMT value is less than 0.85 (Kline, 2011). In summary, discriminant validity for full and split datasets was identified, as seen in Tables 7, 8.

**TABLE 7:** Discriminant validity (Full Dataset).

Construct	Fronell- larcker criterion		HTMT
	Entrepreneurship education	Pre- start-up behavior	Entrepreneurship education
Entrepreneurship education	0.897		
Pre-start-up behavior	0.704	0.870	0.724

**TABLE 8:** Discriminant validity (Split dataset).

Construct	Fronell- larcker criterion				HTMT	
	Business		Non-business		Business	Non-business
	Entrepreneurship education	Pre- start-up behavior	Entrepreneurship education	Pre- start-up behavior	Entrepreneurship education	Entrepreneurship education
Entrepreneurship education	0.9		0.878			
Pre- start-up behavior	0.733	0.885	0.615	0.839	0.752	0.634

Subsequently, standardized root mean square residuals (SRMR) were used to evaluate the goodness of the three models (full and split). An SRMR value of less than 0.08 indicates an acceptable fit. In this study, the SRMR values for the full model were 0.030 and 0.031 for the first group (Business), and 0.045 for the second group (Non-Business), all of which were lower than the suggested value of 0.08, demonstrating a good fit between the empirical and theoretical covariance matrix indicated by the models.

#### Structural Model

The findings of hypothesis testing for the full and split datasets using the bootstrapping procedure with 5,000 resamples are shown in Tables 9, 10. On both the full and split datasets, the findings show that entrepreneurship education has a positive and significant effect on pre-start-up behaviors (business and non-business). Thus, it can be concluded this analysis supports H1.

**TABLE 9:** Structural model assessment (Full dataset).

	Path	Std beta	SE	*t*-Value	R^2^	Q^2^
H1	Entrepreneurship education → Pre-start-up behavior	0.704	0.03	23.755	0.495	0.354

**TABLE 10:** Structural model assessment (Split dataset).

	Path	Business					Non-business				
		Std beta	SE	*t*-Value	R^2^	Q^2^	Std beta	SE	*t*-Value	R^2^	Q^2^
H1	Entrepreneurship education → pre-start-up behavior	0.733	0.042	17.547	0.537	0.390	0.615	0.045	13.635	0.378	0.246

The next step in assessing the structural model’s quality was to calculate the R^2^ values for endogenous constructs as indicators of the models’ explanatory powers. Pre-start-up behaviors have an R^2^ value of 49.5% for the entire dataset, 53.7% for business students, and 37.8% for non-business students, as shown in Table 8. To achieve the minimum level of explanatory power, Falk and Miller (1992) suggest that R^2^ is greater than 0.10. Thus, all endogenous constructs have explanatory power for both full and split datasets.

Finally, using the blindfolding technique, the predictive relevance of all datasets (Q^2^) was evaluated, as shown in Tables 9, 10. Both datasets (full and split) had Q^2^ values greater than zero for entrepreneurship education and pre-start-up behavior, confirming the predictive relevance of all models.

#### Measurement Invariance

This study used the measurement invariance of composite models (MICOM) to compare the outcomes of business and non-business courses in terms of their entrepreneurship education toward pre-start-up behavior. The primary goal of this test was to ensure that both groups interpreted the measurements in the same way. Furthermore, before conducting a multi-group analysis (MGA), this procedure must be followed. MICOM procedures depend on latent variable scores. These latent variables are interpreted as composites in PLS-SEM, which are linear combinations of indicators, and the PLS-SEM algorithm estimates the indicator weights.

The MICOM procedure consists of three steps: (i) configure invariance assessment (both groups’ measurement models have the same basic factor structure); (ii) compositional invariance assessment (composite scores are not significantly different between groups); and (iii) composite mean values and variances are equal. When configurable and compositional variances are defined, partial measurement invariance is confirmed, and the path coefficients between the two groups can be compared. If partial measurement invariance is defined, and the composite has the same mean values and variance across all groups, the composite is considered valid. Therefore, a full measurement invariance was created.

Then, with a 5,000 resample and two-tail test, the PLS-algorithm and PLS-permutation procedures were carried out. The results can be seen more clearly in Tables 11, 12. First, configural invariance is established because the measurement models have the same factor structure for all constructs across business and non-business students. Next, compositional invariance was also confirmed because the composite scores for all constructs were equal across the two groups. The permutation test indicates that none of the correlation values are significantly different from one another. Finally, equality of mean value and variance was assessed across the two groups. Table 11 shows the partial measurement invariance, which is a major requirement before executing the MGA, based on MICOM data.

**TABLE 11:** Measurement invariance result using permutation test.

Construct	Compositional invariance correlation = 1			Partial measurement invariance established	Equality of measures		Equality of variances		Full measurement invariance established
					
	Configure invariance	C = 1	95% C1		Difference	Confidence interval 95%	Difference	Confidence interval 95%	
Entrepreneurship education	Yes	1	0.999	Yes	0.513	[−0.171, 0.152]	0.292	[−0.175, 0.173]	No
Pre-start-up behavior	Yes	1	0.999	Yes	0.552	[−0.162, 0.147]	0.440	[−0.199, 0.188]	No

**TABLE 12:** Assessment of group difference.

Hypotheses	Relationship	Std beta value		SE value		*t*-value		Path coefficient difference	*P*-value	Permutation	Supported
		Business	Non-business	Business	Non-business	Business	Non-business		Henseler MGA		
H2	Entrepreneurship education pre-start-up behavior	0.734	0.617	0.041	0.040	18.010	13.742	0.118	0.023	0.030	Yes

#### Multi-Group Analysis

Using Henseler’s MGA and the permutation approach, PLS-MGA was used to discover the difference. As shown in Table 12, the MGA output demonstrates significant differences between business and non-business students at 0.05 and 0.01 of the effects of entrepreneurship education on pre-start-up behavior. Furthermore, the significance of the differences in the data was confirmed by both Henseler’s MGA and the permutation approach, supporting the research findings. Therefore, H2 is supported by this analysis.

## Discussion

The results indicate that students have a moderate level of entrepreneurship education. Indirectly, this findings indicate that entrepreneurship education has a positive impact on students’ self -development towards entrepreneurship. Nabi et al. (2017) and Almahry et al. (2018) both claim that entrepreneurship education benefits entrepreneurs and aspiring entrepreneurs by providing them with entrepreneurial knowledge and skills. Students are able to identify business ideas, according to a detailed analysis of the mean score of entrepreneurship education. Innovation is capable of transforming ideas into opportunities that can be commercialized (Blundel et al., 2018; Saenz et al., 2019). This can also distinguish between individuals who are entrepreneurial traits and those who aren’t. As a result, entrepreneurship education at the university level can improve students’ capacity to detect opportunities, gather knowledge, and develop skills that will enable them to take advantage of entrepreneurial opportunities and expand their horizons.

The study also discovered that risk control is a crucial component of entrepreneurship education that should be reinforced. This is due to the findings demonstrating students’ capacity to calculate the risk of obtaining a low mean score. Aspiring entrepreneurs must be more daring when calculating risks and seizing potential chances due to challenging business scenarios and an unpredictable economy. According to Mamun et al. (2017) and Ward et al. (2019), a low level of risk-taking and self-efficacy will prevent students from starting a firm. This shows that students who are scared to take risks will not pursue entrepreneurship. They must have faith in their abilities to execute a task despite a number of challenges. They should be confident in whatever decision is made. Thus, aspiring entrepreneurs must be prepared to adapt to any changes that may occur.

Besides that, the study’s findings reveal that students’ behavior in the pre-start-up is moderate. Students who are interested in entrepreneurship will be able to perform entrepreneurship-related activities on their own. Kautonen et al. (2015) and Kong et al. (2020) both agree that the more entrepreneurial activities a person engages in, the greater his or her potential to become an entrepreneur. The detailed findings of the mean score for pre-start-up behavior revealed that respondents were seeking information as well as identifying prospective business opportunities. They have put in the time and effort to start a business. The amount to which a person’s behaviors can impact their actions to embark into entrepreneurship determines a person’s entrepreneurship success (Bird et al., 2012; Reynolds, 2016). This means that someone who wants to thrive in entrepreneurship must set clear goals, recognize opportunities, and move quickly before it’s too late. Therefore, this study shows that entrepreneurial actions or deeds reinforce the behaviors displayed during the business start-up process.

This study aimed to determine the effect of student programs on pre-start-up behaviors. More specifically, this study examined the difference between business and non-business students in terms of their pre-start-up behavior in the Malaysian context, and investigated the relationships between entrepreneurship education and pre-start-up behaviors. The findings of this study confirm the positive effect of entrepreneurship education on students’ pre-start-up behaviors and on the engagement in entrepreneurial activities. This is in line with the human capital theory that students who are exposed to entrepreneurship education may improve their entrepreneurial abilities and thus be more successful in entering entrepreneurship. As a result, in the implementation of entrepreneurship education, curriculum content that supports the formation process of entrepreneurial thinking orientation toward identifying entrepreneurial ideas and opportunities in a creative and inventive way is crucial.

Indirectly, the findings of the study show that entrepreneurship education plays an important role in improving students’ ability to start their own businesses. This is supported by Moreno et al. (2019) and Gieurea et al. (2020) that students’ ability to perform business activities greatly influences them to start a business. This proves that students will be more confident in choosing an entrepreneurial career once they have been exposed to entrepreneurship education. As a result, providing students with entrepreneurial knowledge can help them become more conscious of the field. Furthermore, an entrepreneur’s capacity to discover, understand, and capitalize on opportunities is critical to their success (Kuckertz et al., 2017; Lundmark et al., 2017). Thus, any aspiring entrepreneur must be aware of the entrepreneurial opportunities that exist around them and take those possibilities before others do.

Next, this study compares the effect of entrepreneurship education between business and non-business students on their pre-start-up behaviors. The MGA results showed significant differences between student behaviors in the pre-start-up among business and non-business students. The results suggest that business students scored higher than non-business students in these relationships. This could have occurred as a result of the knowledge and skills gained through entrepreneurship education, which increased their capacity and motivation to start their own businesses. This is consistent with Jones et al. (2017) and Mason et al. (2019) who found that students who have taken an entrepreneurship module or course at university are more likely to pursue an entrepreneurial career than those who did not. Engineering students also assessed themselves less favorably on several personal attributes relevant to entrepreneurship, engaged less with mentors, and were less active in entrepreneurial activities (Jin et al., 2015). Indirectly, this circumstance demonstrates that students who are exposed to diverse entrepreneurship courses or syllabi are more likely to start new businesses.

Furthermore, the results demonstrated that the effect of entrepreneurship education on pre-start-up behavior is greater for business students (*B* = 0.734) than for non-business students (*B* = 0.617), indicating that H2 is supported. This finding clearly shows that providing students with entrepreneurship education can help them become more efficient in their entrepreneurial endeavors. This means that entrepreneurship education not only encompasses entrepreneurship but also the process of developing students’ entrepreneurial abilities. In addition, an entrepreneur’s success is determined by the extent to which entrepreneurial behavior is embraced. This implies that entrepreneurship does not exist without action. Hence, individuals who are actively involved in the pre-start-up activities are also more likely to launch a business (Reynolds, 2016; Mamun et al., 2017; Li and Wu, 2019). This may be shown in their efforts, time, and money spent prior to starting the business. Therefore, entrepreneurship education must be properly designed, and the co-curriculum must be properly organized so that entrepreneurship will be the preferred career choice in the future.

## Implications

Overall, this study contributes to the body of knowledge on the relationship between entrepreneurship education and pre-start-up behavior. Indirectly, these findings may aid the human capital theory in the development of students’ entrepreneurial potential. Thus, it is no surprise that many scholars have accepted human capital theory, which views entrepreneurship education as an investment. Therefore, the outcome of entrepreneurship education acquired by students, particularly at the tertiary level, may be used to assess the quality of human capital developed.

Practically, the results of this study provide a clear insight into the engagement of different groups of student programs in the pre-start-up behaviors. Findings indicate that students in business programs who have a proclivity for entrepreneurship will take early steps before establishing a business. Indirectly, the more entrepreneurial activities a student engages in during the business start-up process, the greater his or her chances are of becoming an entrepreneur. Thus, at the tertiary level, exposure to entrepreneurial knowledge and experience may result in the development of quality human capital that is inventive, skilled, and competitive. This is consistent with the National Entrepreneurship Policy’s aim of transforming Malaysia into an entrepreneurial nation by 2030.

In addition, this study has several practical implications. In order for entrepreneurship education to be implemented effectively in the university, entrepreneurship instructors must be kept updated to the most relevant and effective approaches to educate their students. In comparison to “traditional” teaching, students require more efficient entrepreneurship education such as business simulation and case studies. Furthermore, risk control elements must be addressed by a university’s curriculum committee. This element can be cultivated across the curriculum, not just in entrepreneurship courses or subjects. Indirectly, students are better equipped to manage risks, particularly in business. They must be willing to take chances as well as have a confidence and determination to succeed in business. Next, the university can appoint mentors from among successful entrepreneurs or alumni to help students interested in entrepreneurship manage their businesses successfully. This can help boost the entrepreneurial ecosystem by implementing comprehensive entrepreneurship education and supporting students who want to start their own businesses.

## Limitation and Future Research

This study only involved respondents who were students in public universities in Malaysia. Thus, future researchers should broaden the scope of the study to include private universities and explore comparisons across developing countries. Indirectly, future studies could add new information into the literature based on the context of entrepreneurship education with pre-start-ups in developing countries. Besides that, the study of entrepreneurship education on pre-start-up behavior is still novel as previous research has paid little attention to the subject. In the future, new constructs will hopefully be developed to examine the components of entrepreneurship education outcomes, such as attitude and personality.

## Conclusion

In today’s world, entrepreneurship education is significant for preparing students to become entrepreneurs at the tertiary level. Students will be more inclined to engage in entrepreneurial activities toward the establishment of a business if they have a good impression of their own capabilities. This indicates that there will be no entrepreneurship if no entrepreneurial activity is conducted, as it is the starting point of a business. The findings prove that students with entrepreneurial experience and expertise are more likely to perceive new possibilities and possess the resources needed to take advantage of these opportunities. Furthermore, in a competitive market, the unpredictable and more difficult economic environment requires students and would-be entrepreneurs to be more innovative, viable, and resilient. Additionally, the entrepreneurial behaviors of developing countries are influenced by their cultural norms. The way entrepreneurs run their businesses is influenced by many cultural beliefs and their thinking. Thus, the university should provide opportunities for all students to pursue entrepreneurship education in order to develop awareness and ultimately select an entrepreneurship career.

## Data Availability Statement

The original contributions presented in the study are included in the article/supplementary material, further inquiries can be directed to the corresponding author/s.

## Author Contributions

NHO, NO, and NHJ contributed to design of the study. NHO organized the database and performed the statistical analysis. NHO wrote the first draft of the manuscript. All authors contributed to manuscript revision, read, and approved the submitted version.

## Conflict of Interest

The authors declare that the research was conducted in the absence of any commercial or financial relationships that could be construed as a potential conflict of interest.

## Publisher’s Note

All claims expressed in this article are solely those of the authors and do not necessarily represent those of their affiliated organizations, or those of the publisher, the editors and the reviewers. Any product that may be evaluated in this article, or claim that may be made by its manufacturer, is not guaranteed or endorsed by the publisher.

## Appendix 1

**Table:** Tabular Form of Literature Reviews.

Author	Year	Sample	Findings
Gieurea et al., 2020	2020	Students	This research shows that when persons have the relevant skills, they have a positive attitude toward beginning a business. When students have the knowledge or capacity to start a business, their attitudes regarding beginning a business will be exposed.

Jones et al., 2017	2017	Gradutes students	The research shows that through the enterprising knowledge and skill sets graduates gain during their specialised studies, EE programmes bring value both in terms of enabling business start-ups and in supporting other career pathways.
Lee et al., 2018	2018	Students	Networking, proactive, and self-confidence skills are identified as entrepreneurial abilities that may be taught to students through an entrepreneurship education programme. Students in a business department have much poorer entrepreneurial abilities than students in an engineering programme.
Li and Wu, 2019	2019	Students	Entrepreneurship education enhances students’ readiness to start a business and contributes to an understanding of why and how entrepreneurial education boosts the entrepreneurial intentions of business students.
Lundmark et al., 2017	2017	Students	The findings demonstrate that entrepreneurship education reflections help students develop their entrepreneurial skills and discovered that starting a business helps students enhance entrepreneurial skills.
Mamun et al., 2017	2017	Students	The findings indicate a positive and significant relationship between entrepreneurial intent and student preparation for entrepreneurship. Indeed, this research demonstrates the importance of government support, family support, and entrepreneurship development programmes in influencing student behavior.
Othman et al., 2020	2020	Students	The findings show that students’ actions to seize opportunities are important and are influenced by their exposure to entrepreneurial learning.
Saenz et al., 2019	2019	Students	Students with high entrepreneurial abilities on four dimensions (capacity, planning, creativity, dedication, and responsibility), as well as those with good entrepreneurial potential, are more likely to engage in entrepreneurship, according to the findings.
Sanchez and Sahuquillo, 2018	2018	Students	The findings show that future engineers’ need for independence is a crucial element in their entrepreneurial intent, confirming that entrepreneurship education has a favourable impact on their entrepreneurial intentions.
Wardana et al., 2020	2020	Students	Entrepreneurship education can help students develop their entrepreneurial potential while also improving their knowledge and skills. The findings indicate that entrepreneurship education has an impact on students’ entrepreneurship self-efficacy.
